# Exploring the Effect of Age on the Reproductive and Stress Physiology of *Octopus bimaculoides* Using Dermal Hormones

**DOI:** 10.3390/ani13193115

**Published:** 2023-10-06

**Authors:** Stephanie Chancellor, Bret Grasse, Taylor Sakmar, David Scheel, Joel S. Brown, Rachel M. Santymire

**Affiliations:** 1 San Diego Wildlife Alliance, San Diego, CA 92101, USA; 2 Marine Biological Laboratory, Woods Hole, MA 02543, USA; bgrasse@mbl.edu (B.G.); tsakmar@mbl.edu (T.S.); 3 Institute of Culture and the Environment, Alaska Pacific University, Anchorage, AK 99508, USA; dscheel@alaskapacific.edu; 4 Department of Integrated Mathematical Oncology, Moffitt Cancer Center, Tampa, FL 33612, USA; Joel.Brown@moffitt.org; 5 Biology Department, Georgia State University, Atlanta, GA 30302, USA; rsantymire@gsu.edu

**Keywords:** California two-spot octopus, cortisol, estradiol, progesterone, senescence, testosterone

## Abstract

**Simple Summary:**

Octopuses are charismatic animals commonly kept in aquaria and increasingly used for scientific research. As we begin to care for more of these animals in captivity, an understanding of how they perceive life in an aquarium is necessary to provide adequate welfare. Further, octopuses have a comparably short life span, averaging about 1–2 years. Due to these factors, it is necessary to learn more about how to care for these animals and increase their reproductive output while living in an aquarium. In this study, we validated a noninvasive technique to measure the glucocorticoids and reproductive hormones of the California two-spot Octopus, *Octopus bimaculoides*. We tested for differences in these hormones between reproductive and senescent (animals towards the end of their life) individuals. Reproductive individuals had higher sex hormone concentrations than senescent individuals, while the reverse was true for stress hormones. In terms of their stress response, reproductive individuals had a higher increase in glucocorticoids concentrations compared to their senescent counterparts after being exposed to a stressful event. These results provide a first step in broadening our ability to noninvasively collect hormones and expand our knowledge of how octopus hormones change throughout their lifetime.

**Abstract:**

Our goal was to validate the use of dermal swabs to evaluate both reproductive and stress physiology in the California two-spot octopus, *Octopus bimaculoides*. Our objectives were to (1) use dermal swabs to evaluate glucocorticoids and reproductive hormones of *O. bimaculoides*; (2) determine the influence of life stage on hormone production (glucocorticoids in all individuals; testosterone, estrogen, and progesterone in females; and testosterone in males) of reproductive (*n* = 4) and senescent (*n* = 8) individuals to determine the effect of age on hormonal patterns; and (3) determine whether these hormones change significantly in response to an acute stressor. For the stress test, individuals were first swabbed for a baseline and then chased around the aquarium with a net for 5 min. Afterward, individuals were swabbed for 2 h at 15 min intervals to compare to the pre-stress test swab. Reproductive individuals responded to the stressor with a 2-fold increase in dermal cortisol concentrations at 15 and 90 min. Six of the eight senescent individuals did not produce a 2-fold increase in dermal cortisol concentrations. Reproductive individuals had significantly higher sex hormone concentrations compared to senescent individuals (progesterone and estradiol measured in females, and testosterone for both sexes). After the stressor, only reproductive males produced a 2-fold increase in dermal testosterone concentrations, while sex hormones in females showed no change. The stress hormone cortisol was significantly higher in senescent than in reproductive individuals, independent of sex. Dermal corticosterone concentrations were highest in senescent females followed by senescent males, and lowest in reproductive individuals regardless of sex. Dermal swabs provide an effective and noninvasive means for evaluating octopus hormones. Application of these indicators may be imperative as cephalopods are more commonly cultured in captivity for experimentation, display, and consumption.

## 1. Introduction

Aquaculture of cephalopods is important for providing a sustainable population for public aquariums, advancement of our understanding of these behaviorally complex organisms through scientific research, and food production. However, we need to improve our understanding of how living in an ex situ environment affects this animal group’s physiology. This can be challenging because of the diverse life histories found among cephalopod species.

Cephalopods live in a variety of habitats throughout the ocean, from the deepest depths to the shallow shores [1]. They also possess the ability to change the texture and color of their skin [2,3,4,5] and to solve problems [6,7]. Although they tend to be regarded as intelligent animals, cephalopods have rather short life spans, on average living approximately 1.5–2 years [8]. Almost all octopuses are semelparous [8,9], reproducing once in their life before a swift and fatal decline in health.

In general, cephalopods invest in growth during the development phase of their life [9,10]. Growth and sexual maturity are regulated by secretions into the blood from the optic gland [11], which is an analog of the vertebrate pituitary gland [12]. The optic gland helps regulate behavior through numerous hormonal pathways [12] including sexual maturity, which is signaled by a hormone analogous to gonadotrophin (GnRH), called oct-GnRH [13]. Oct-GnRH stimulates and regulates the synthesis and release of sex steroids (testosterone, estradiol-17β, and progesterone). These sex hormones are present in the sex organs and blood of octopuses [14]. While laying eggs, females will restrict growth and devote all their resources to their ovaries [15]. Specifically, estradiol and progesterone increase during vitellogenesis, or yolk development [16]. In males, testosterone stimulates spermiogenesis and reproductive behaviors [14,17].

In semelparous species of octopus, after egg laying, female senescence is controlled by the activation of the optic gland and oct-GnRH [12,15]. Males, whether they mate or not, experience a similar decline in health around the same age as when females lay their eggs [15]. Senescence can happen rapidly [5,18] as individuals exhibit a decline in feeding, retraction of skin around the eyes, uncoordinated movement, lesions on the body, and increased and undirected activity [8]. Additionally, senescing females typically are brooding eggs, while males become more active in search of mates [5,8].

Like reproductive hormones, glucocorticoid production varies throughout an animal’s life span due to life events and metabolic differences between life stages [19,20,21,22,23]. The Hypothalamic–Pituitary–Adrenal (HPA) axis activation is well known in vertebrates. An animal’s encounter with a perceived stressor results in the hypothalamus releasing the corticotropin releasing hormone, which stimulates the anterior pituitary gland to release the adrenocorticotropic hormone (ACTH) [24,25]. Then, ACTH stimulates the adrenal glands to release glucocorticoids, including cortisol and corticosterone. These steroids cause an increase in the release and production of glucose.

In vertebrates, the complex HPA axis can be influenced by several factors such as environment, age, or past experiences [23,26]. The stress-physiology pathways of cephalopods are unknown [27]. Yet, research has shown that glucocorticoids in octopuses respond to stressors or changes in homeostasis similarly to vertebrates [27,28]. For example, juvenile cephalopods produce a higher concentration of glucocorticoids compared to older individuals [28], whereas in vertebrates, glucocorticoids can vary in concentration and function throughout life stages [23]. Both glucocorticoids have been measured in cephalopods, cortisol was found to be the more biologically informative glucocorticoid in cephalopod dermal secretions [28].

Understanding octopus endocrinology contributes to comprehending cephalopod physiology and life history, and it provides insights for diagnosing health issues and alleviating the stresses of captivity as culturing [29] and octopus farming [30] become more common. The inability to sample individuals noninvasively poses a challenge for studying cephalopod endocrinology. Previous research has euthanized individuals to analyze changes in reproductive hormones at different life stages by harvesting their reproductive organs [14,16]. Other studies used blood draws to measure glucocorticoids [31]. The advantage of noninvasive techniques for measuring hormones is that they allow for repeated sampling of the same individual. While reproductive hormones and glucocorticoids have been measured noninvasively through feces [28], due to water circulation and group cultures in aquaria, it can be difficult to collect feces in a standardized manner without constant monitoring. Collecting mucus via swabs from the octopus’ skin surface may provide a noninvasive alternative for measuring hormones. Dermal swabs have been used previously in fish [31,32,33,34,35,36] and amphibians [36] and were recently validated for use with three cephalopod species, *Euprymna berryi*, *Sepia bandensis*, and *Octopus chierchaie*, [28].

Collecting dermal mucus swabs throughout the entirety of an individual cephalopod’s life span could determine normal hormonal changes across age and life stages. For example, understanding changes in hormones may help determine if an individual is in poor health from environmental factors, illness, or if it is experiencing natural senescence. Until recently, breeding cephalopods in aquaria and laboratory settings has been difficult, and for some species remains impossible. It is difficult to age wild-caught individuals because most indicators of age are internal. Additionally, indicators of senescence can be like those of various diseases or infections in cephalopods. This makes it difficult to distinguish whether the animal is experiencing a natural decline or has a treatable illness [18]. Further, senescence itself can look like an animal welfare issue, as the animal’s health collapses. Whether to euthanize or allow senescent octopuses to live out their life remains an open question in animal welfare [37]. Understanding how glucocorticoids and reproductive hormones change throughout an individual’s lifespan may prove helpful when their determining age and health status.

Our goal is to expand the knowledge of cephalopod endocrinology by using dermal mucus swabs to measure glucocorticoid and reproductive hormones in the reproductive and senescent life stages in the California two-spot octopus (*Octopus bimaculoides*). *O. bimaculoides* was the first cephalopod species to have its genome sequenced [38] and has since been used extensively in research [39]. The lifespan of *O. bimaculoides* is 15–17 months. Their life history includes three stages: juveniles [40], reproductive adults, and post-reproductive senescent adults [41]. Death typically occurs within 2 months after egg spawning [42]. Males senesce around the same time as females [8]. Upon reaching adulthood, even in the absence of opportunities to mate, *O. bimaculoides* will senesce in 12–14 months.

Our objectives are to (1) use dermal swabs to evaluate glucocorticoids and reproductive hormones of *O. bimaculoides*; (2) determine the influence of life stage on hormone production; and (3) determine whether these hormones change significantly in response to an acute stressor. We predict that senescent individuals will produce lower concentrations of dermal glucocorticoids and reproductive hormones than reproductive individuals, as their health declines. We expect a larger and more immediate increase in dermal glucocorticoid concentrations than concentrations of reproductive hormones in response to an acute stressor. We also predict a difference in dermal glucocorticoid concentrations between reproductive adults and senescent adults, as glucocorticoids have different roles throughout the life stages of an animal [23].

## 2. Materials and Methods

### 2.1. Ethical Statement, Study Site, and Subjects

In the United States cephalopods are not regulated by the animal care and use protocol. While not necessary to apply for IACUC approval, we followed methods established by the regulations of Directive 2010/63/EU for cephalopods [37,43,44]. The research was approved by Lincoln Park Zoo’s Research Committee (approval #2018-016).

Study animals were individually housed in the mariculture room of the Marine Biological Laboratory in Woods Hole, MA, USA. *O. bimaculoides* was chosen due to its availability and its increasing use in research since the publication of their genome [38]. Animals were wild caught from the California coast using the pot fishing method, and then sent to the Marine Biology Laboratory via overnight mail. Individuals were either reproductive adults (2 males and 2 females) or senescent adults (3 males and 5 females). Upon the initiation of this study, senescence was determined through common indicators. In males, we monitored for curling of the tips of their arms, development of lesions, lack of eating, and/or when they died, naturally, in relation to the study timing. Females were considered to be senescent after their eggs were laid. The time between receiving the females and laying of eggs varied by individual. Senescent females were removed from their eggs hours before data collection. Octopus enclosures included material to den in, such as a terra cotta pot, but otherwise, enclosures were bare. Each enclosure contained 5 gallons of seawater and was part of a larger semi-open system, housing numerous octopuses with a flow of approximately 0.4 L/min. Octopuses had been acclimated to these aquaria for 2–3 months before initiating this study. All were maintained on a 12:12 h light:dark schedule. They were fed a diet of live crabs.

### 2.2. Swabbing Method

Octopuses remained submerged in their aquarium during swabbing to minimize additional stress caused by the procedure. Octopuses were not restrained when swabs were taken. Swabs were collected in the afternoon. While wearing powder-free latex gloves, a sterilized cotton swab with a plastic handle was dipped into the aquarium and rubbed back and forth 4 times over approximately 2.5 cm of the mantle of each individual. Swabs were then placed in a 2.0 mL tube containing 1 mL of 70% ethanol:distilled water. To ensure that the tube was sealed properly, swab handles were cut to fit into the 2.0 mL tube, and parafilm was wrapped around the lid to prevent leakage or evaporation. After collection, swabs were stored in a −32 °C freezer.

### 2.3. Validation of Glucocorticoid Analysis Using a Stress Test

Prior to beginning the stress test, individuals were swabbed to obtain a baseline sample of dermal hormones. For the stress response, we disturbed each animal with a small net ensuring each individual moved throughout the aquarium for 5 min. Following the stress test, octopuses’ mantles were swabbed while they remained in their aquaria, underwater and unrestrained. Then, individuals were swabbed every 15 min for the next 2 h, which produced 10 data points per individual. Samples were analyzed for hormone concentrations using the methods described below.

### 2.4. Sample Processing and Hormonal Analysis

We processed all samples at the Davee Center for Epidemiology and Endocrinology (Lincoln Park Zoo, Chicago, IL, USA) using previously described methods [28]. Briefly, samples were mixed for 5 min in a mixer (Glas-col, Terre Haute, IN, USA; setting 60–70 rpm). Then, 500 µL of ethanol from each sample was pipetted into a new, pre-labeled 15 × 75 mm test tube. Under forced air in a warm water bath (60 °C), aliquots were dried down and 2–3 glass beads were added to each tube, along with 500 µL of phosphate-buffered saline (PBS; 0.2 M NaH_2_PO_4,_ 0.2 M Na_2_HPO_4_, NaCl). We briefly vortexed tubes and then sonicated them for 20 min. Finally, for 30 min, samples were shaken again on the Glas-col mixer (60–70 rpm) and stored at 5 °C until they were analyzed using an enzyme immunoassay (EIA).

We used a previously described cortisol EIA to measure cortisol concentrations with dermal swabs [35,36]. Briefly, the polyclonal cortisol antiserum (R4866) and horseradish peroxidase (HRP), provided by C. Munro (University of California, Davis, CA, USA), were used and diluted to 1:375,000 and 1:200,000, respectively, in a 96-well plate that was coated with goat anti-rabbit antibody (1:1000, Arbor Assay, Ann Arbor, MI, USA). The cross-reactivity of cortisol antibody has been previously reported [45,46]. The cortisol EIA was biochemically validated by demonstrating parallelism between binding inhibition curves of swab hormones (2 × concentrated to 1:16) and the cortisol standard (r = 0.993) and significant recovery of cortisol added to pooled ethanol from swab samples (y = 1.100 × −6.422, R^2^ = 0.993, *p* < 0.001). Assay sensitivity was 1.95 pg/well and intra- and inter-assay coefficients of variation were <15%.

Progesterone polyclonal antiserum (CL425; University of California, Davis, CA, USA) and HRP were used at a dilution of 1:70,000 and 1:1,000,000, respectively, and used on similarly prepared plates as described above, except we used a secondary goat anti-mouse immunoglobulin G (IgG) (A008, Abor Assays, Ann Arbor, MI, USA) as described by Glaeser et al. [47]. Cross-reactivities of the progesterone antibody were previously reported [45,48]. We biochemically validated the progesterone EIA by demonstrating the parallelism between binding inhibition curves of swabs and the progesterone standard (r = 0.995); the significant recovery of exogenous progesterone (0.78–200 pg/well) added to swabs (y = 0.910 × −0.511, R^2^ = 0.999; *p* < 0.001).

Estradiol polyclonal antiserum (R0008; University of California, Davis, CA, USA) and HRP were used at a dilution of 1:250,000 and 1:200,000, respectively. We used the same prepared plates as the cortisol EIA. Cross-reactivities of the estradiol antibody were previously reported [49]. We biochemically validated the estradiol EIA by demonstrating the parallelism between binding inhibition curves of swabs and the estradiol standard (r = 0.994); and the significant recovery of exogenous estradiol (1.95–250 pg/well) added to swabs (y = 0.999 × −3.461, R^2^ = 0.998; *p* < 0.001).

Testosterone horseradish peroxidase (HRP) ligands and polyclonal antiserum (R156/7; University of California, Davis, CA, USA) were used at dilutions of 1:750,000 and 1:375,000, respectively, on the same prepared plates as the cortisol and estradiol EIAs. Antiserum cross-reactivities for testosterone were previously reported [50]. We validated the testosterone EIA by demonstrating the parallelism between binding inhibition curves of swabs and the testosterone standard (r = 0.975); and the significant recovery of exogenous testosterone (1.17–300 pg/well) added to swabs (y = 1.035 × −3.784, R^2^ = 0.999; *p* < 0.001).

### 2.5. Data Analysis

For glucocorticoid analysis, a sample was considered elevated after the stress test if glucocorticoid concentrations were at least twice (2-fold higher or 200%) that of the pre-stressor, baseline sample [21]. We consider a value less than that to indicate little response to the stress test. While other researchers have validated the use of dermal swabs to measure changes in glucocorticoids of other aquatic species, such as *Mola mola*, using a 1.5-fold increase or higher as an indicator of a stress response [35], we considered the 2-fold (or 200%) increase to be indicative of a response to a stressor since our understanding of cephalopod stress physiology is limited. Regardless, we report the increase above baseline and invite future consideration of appropriate cut-offs for a demonstrable increase in response to a stressor. To determine the effects of age, sex, and the interaction of the two on reproductive (testosterone, progesterone, and estradiol) and stress (cortisol and corticosterone) hormone concentrations, we used a repeated measures analysis of variance with 10 time points for each individual (rmANOVA) (SYSTAT13). Univariate F-tests tested for effects independent of time, such as age and sex. In the univariate tests, individuals represent the unit of replication. If there were overall effects of time on any of these hormones (after adjusting for experiment-wise error rates), we only considered the linear, quadratic, or cubic terms as biologically meaningful. For females, we tested for the effect of age on the reproductive hormones, progesterone and estradiol. Conditions for normality were met, though this may be due to the small number of individuals and having a single measurement per individual per time point. Given the small number of individuals, all results should be considered as instructive, not definitive.

## 3. Results

### 3.1. Glucocorticoid Analysis

**Figure 1:**
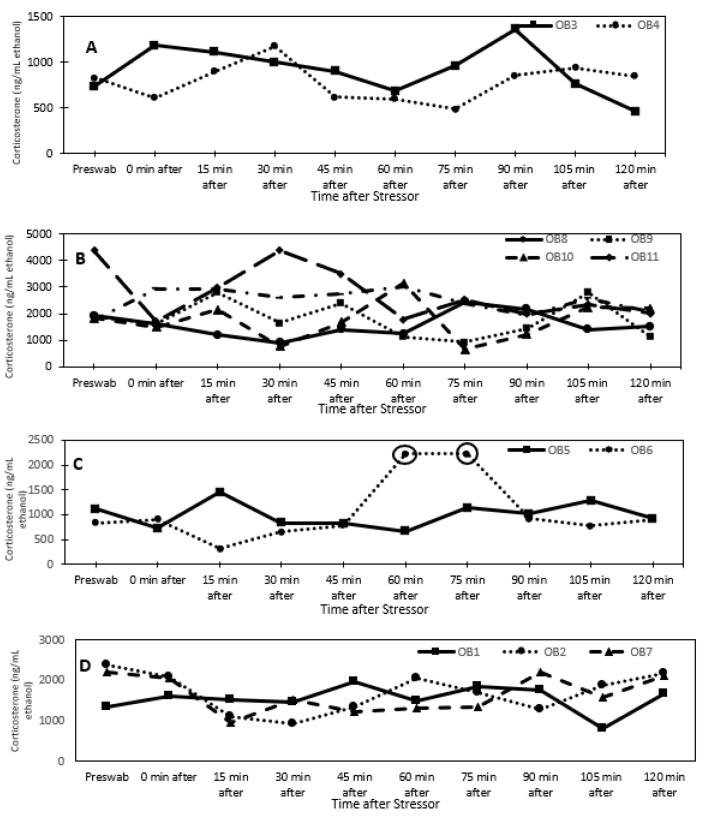
Dermal corticosterone concentrations (ng/mL ethanol) over time after a stress test (chasing octopus around for 5 min with a net) for (**A**) reproductive females, (**B**) senescent females, (**C**) reproductive males, and (**D**) senescent males. The circles indicate an elevated response that is at least 2-fold above the pre-swab sample.

**Figure 2:**
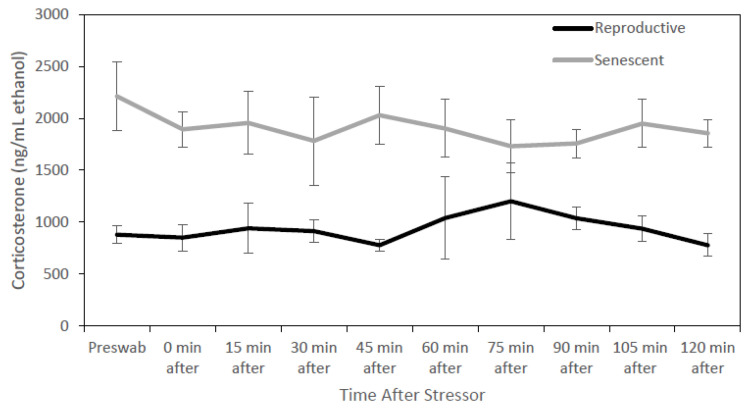
Senescent octopus (8 individuals) had higher (*p* < 0.05) dermal corticosterone concentrations (mean ± SEM ng/mL ethanol) than reproductive individuals (4 individuals) before and after a stress test (chasing octopus around for 5 min with a net). Temporal trends were not significant.

For all individuals, dermal corticosterone concentrations remained below the 2-fold threshold for 2 h after the stressor, except for one reproductive male at times 60 and 75 min after the stress test (Figure 1). For dermal cortisol, a 2-fold increase in concentration was observed in three of the four reproductive individuals after the stressor. Due to this result, corticosterone may not be biologically informative for identifying acute stress in *O. bimaculoides.* In fact, dermal corticosterone levels were higher (univariate analysis: F_1,8_ = 15.84, *p* < 0.005) in senescent (1907.1 ± 161.9 ng/mL ethanol) than in reproductive (935.9 ± 122.6 ng/mL ethanol) individuals (Figure 2). There were no significant effects of sex, sex by age, or time.

**Figure 3:**
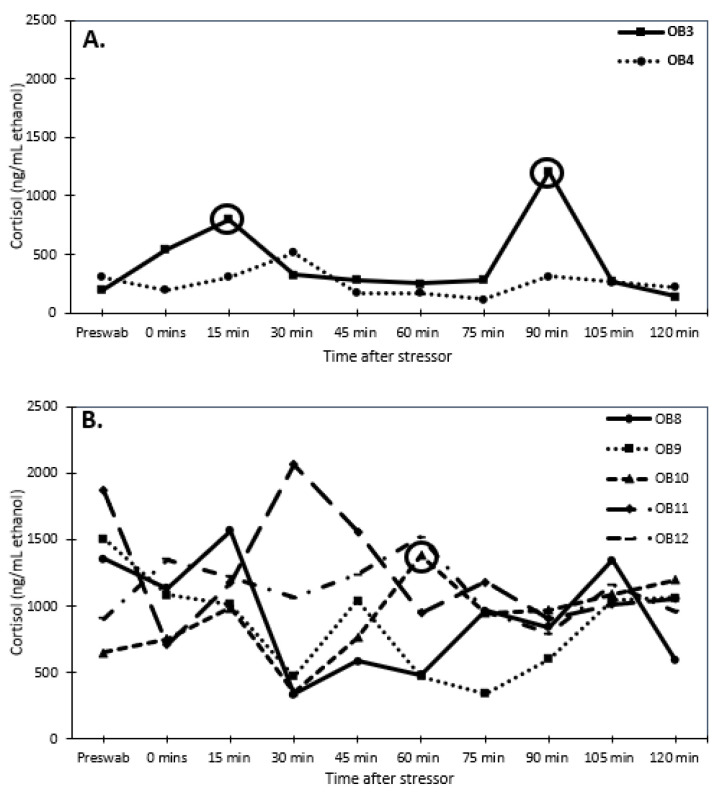
Dermal cortisol concentrations (ng/mL ethanol) over time after a stress test (chasing octopus around for 5 min with a net) for (**A**) reproductive female (2 individuals) and (**B**) senescent female (5 individuals) *Octopus bimaculoides.* The pre-swab serves as a baseline value for each individual. The circles indicate an elevated response of at least 2-fold higher than the pre-swab sample.

**Figure 4:**
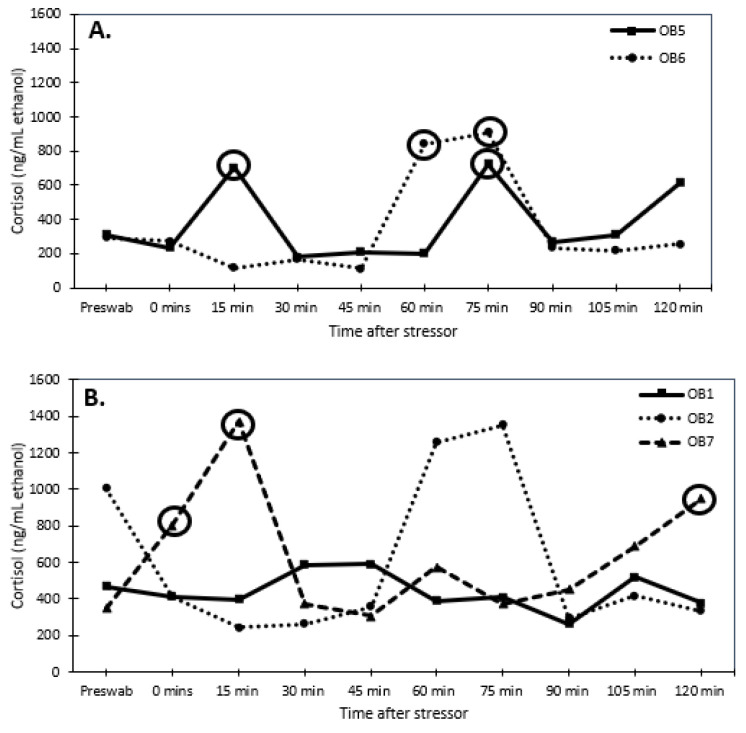
Dermal cortisol concentrations (ng/mL ethanol) over time after stress test (chasing octopus around for 5 min with a net) for (**A**) reproductive male (2 individuals) and (**B**) senescent male (3 individuals) Octopus bimaculoides. The pre-swab serves as a baseline value for each individual. The circles indicate an elevated response of at least 2-fold higher than the pre-swab sample.

Senescent females had considerably higher dermal cortisol concentrations than senescent males (F_1,78_ = 30.947, *p* < 0.001; 1010.8 ± 52.5 and 552.9 ± 57.9 ng/mL ethanol, respectively). Reproductive males and females had similar dermal cortisol concentrations (F_1,38_ = 0.042 *p* < 0.8; 358.8 ± 54.1 and 342.3 ± 56 ng/mL ethanol, respectively). Thus, senescent individuals had higher (univariate analysis: F_1,8_ = 27.2, *p* < 0.001) cortisol concentrations than reproductive individuals (Figure 3 and Figure 4). There was an interaction (univariate analysis: F_1,8_ = 8.23, *p* < 0.025) between age and sex. There were no significant effects of time or interactions of time with sex and age.

When examining changes in dermal cortisol concentrations, one reproductive female (OB3) responded with a 2-fold increase in dermal cortisol concentrations, while the other reproductive female (OB4) experienced a 1.6-fold change, but it did not meet the 2-fold threshold (Figure 3A). None of the senescent females produced a 2-fold increase in dermal cortisol levels after the stressor, except for one female (OB10) at 60 min (Figure 3B). Both reproductive males responded to the stressor with a 2-fold increase in dermal cortisol concentrations (Figure 4A). The senescent males did not produce a 2-fold response in dermal cortisol except for one male (OB7) at 0, 15, and 120 min (Figure 4B).

### 3.2. Effect of Age and Sex on Reproductive Hormones

**Figure 5:**
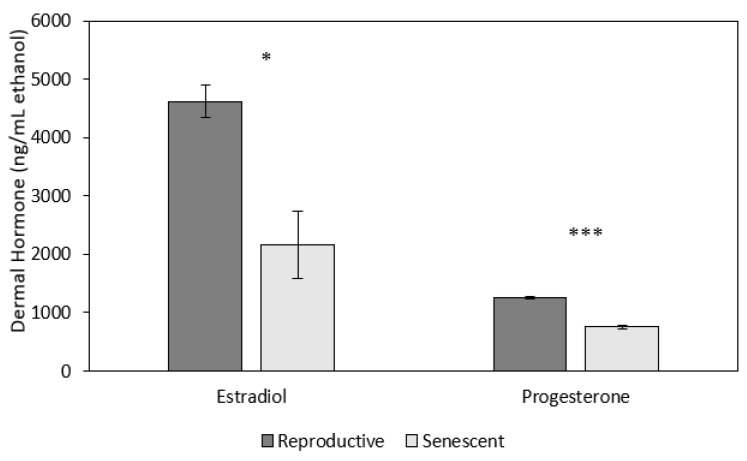
Mean (±SEM) dermal progesterone and estradiol concentrations were significantly higher for reproductive (2 individuals) than senescent (5 individuals) female *Octopus bimaculoides* (10 measurement time points per individual, starting prior to and up to 2 h after the stress test). Asterisks indicate a significant difference (univariate F-tests: F_1,5_ = 7.6, * *p* < 0.05; F_1,5_ = 78.9, *** *p* < 0.001, respectively) between reproductive and senescent females.

Both dermal progesterone (univariate analysis: F_1,5_ = 78.9, *p* < 0.001) and estradiol (univariate analysis: F_1,5_ = 7.6, *p* < 0.05; Figure 5) concentrations were higher in reproductive females (progesterone: 1261.7 ± 39.9; estradiol: 4618.9 ± 575.9 ng/mL ethanol) than in senescent females (progesterone: 753.1 ± 23.0; estradiol: 2162.2 ± 280.4 ng/mL ethanol; Figure 5). There were no significant effects of time or time by age on progesterone or estradiol (Figure 5).

**Figure 6:**
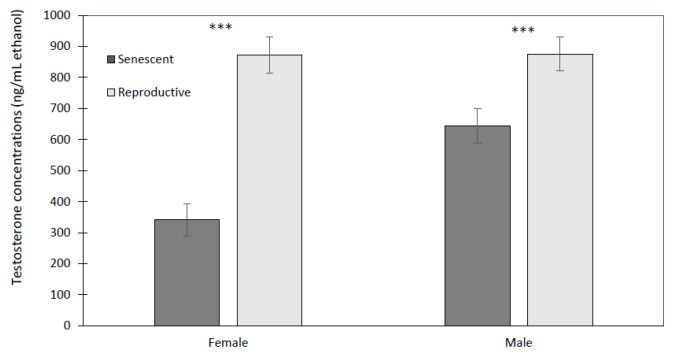
Mean (±SEM) dermal testosterone concentrations were significantly lower for senescent (5 females and 3 males) than reproductive (2 females and 2 males) *Octopus bimaculoides* (10 measurement time points per individual, starting prior to and up to 2 h after the stress test). Asterisks indicate a significant difference (univariate F-test: F_1,8_ = 22.6, *** *p* < 0.001) between reproductive and senescent individuals.

**Figure 7:**
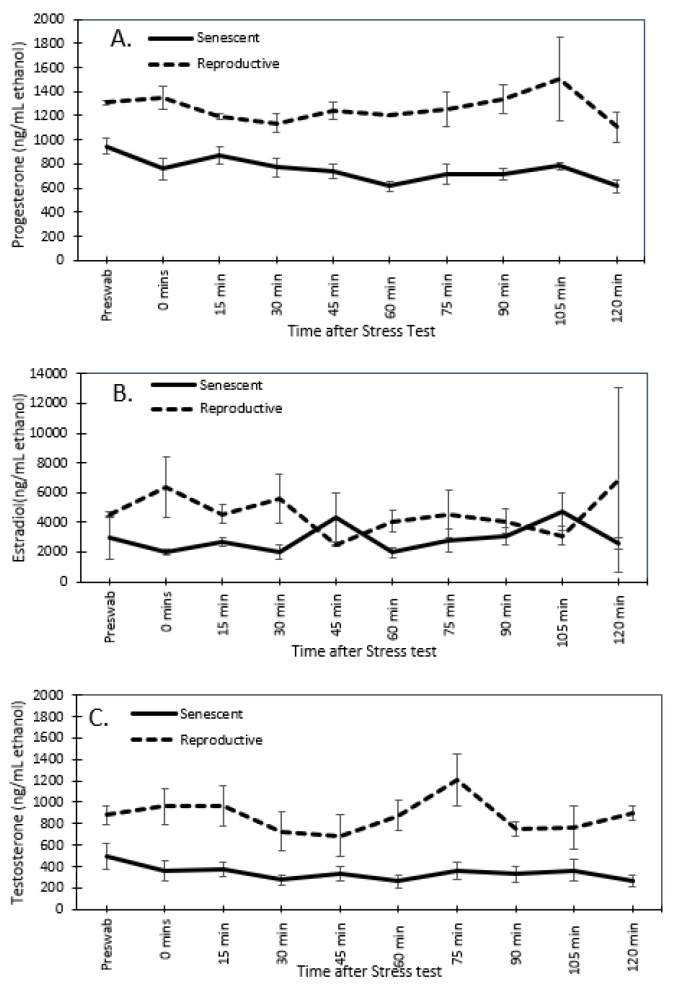
Mean (±SEM) of dermal progesterone (**A**), estradiol (**B**), and testosterone (**C**) for senescent (5 individuals) and reproductive (2 individuals) *Octopus bimaculoides* females from the pre-swab through 2 h after the stress test.

Of all hormones, testosterone showed the most varied effects from sex, age, and time. Reproductive males (875.8 ± 76.3 ng/mL ethanol) and females (872.9 ± 49.4 ng/mL ethanol) had higher concentrations than senescent males (644.6 ± 50.5 ng/mL ethanol) and females (341.4 ± 24.2 ng/mL ethanol) (Figure 6). The effect of age was significant (univariate analysis: F_1,8_ = 22.6, *p* < 0.001) but not so for sex (univariate analysis: F_1,8_ = 3.64, *p* = 0.093) or the interaction of sex with age (univariate analysis: F_1,8_ = 3.50, *p* = 0.098). There were significant time effects but only as the interaction of time with age (multivariate analysis: F_9,72_ = 2.41, *p* < 0.02), and the three-way interaction of time by age by sex (multivariate analysis: F_9,72_ = 2.06, *p* < 0.05). These emerged from significant linear relationships of time with age (polynomial test of order 1: F_1,8_ = 10.33, *p* < 0.02), and time with age by sex (polynomial test of order 1: F_1,8_ = 6.85, *p* < 0.05). Only reproductive males showed a significant increase in testosterone at 75 min post-stressor, while all other groups had relatively constant dermal testosterone levels with time, unaffected by the stressor. Reproductive females maintained a higher dermal testosterone level than senescent females over all time points (Figure 6), whereas it is only at 75 min post-stressor that reproductive males have a significantly higher testosterone level than senescent males (Figure 7).

### 3.3. Effect of the Stress Test on Reproductive Hormones

**Figure 8:**
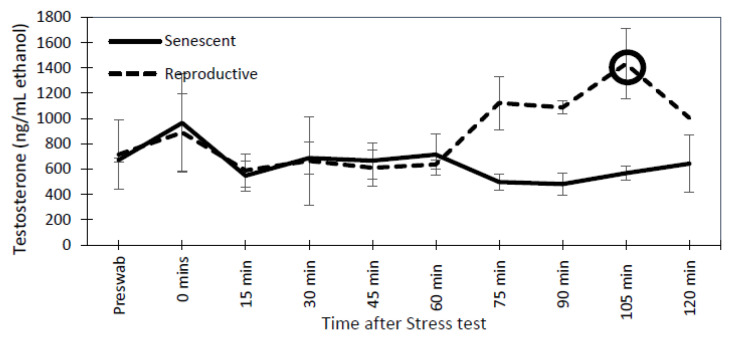
Mean (±SEM) testosterone levels for senescent (3 individuals) and reproductive (2 individuals) *Octopus bimaculoides* males from pre-swab sampling until 2 h after stress test. The circle indicates an elevated response in testosterone that is at least 2-fold above the pre-swab sample.

For reproductive and senescent females, dermal progesterone, estradiol, and testosterone concentrations remained below the 2-fold change threshold over the post-stressor period (Figure 7). Similarly, in senescent males, dermal testosterone concentrations did not have a 2-fold change post-stressor. However, both reproductive males had a 2-fold peak in testosterone concentrations at 105 min post-stressor (Figure 8).

## 4. Discussion

Endocrinological knowledge is crucial for improving husbandry and promoting the reproduction of animals managed under human care. However, current techniques make it difficult to obtain samples noninvasively in aquatic animals, such as cephalopods. Here, we successfully measured glucocorticoids and reproductive hormones in the common, ex situ reared *O. bimaculoides* using a noninvasive dermal swab technique. Additionally, we used a known stressor, chasing octopuses with a net for 5 min, to test for a biological stress response. This type of procedure has been used to validate stress physiology analyses in other species [23]. Here, this biological stressor invoked elevated cortisol (a 2-fold increase in cortisol from the pre-stressor sample) in both reproductive males (at 15 and 75 min) and one of two reproductive females (at 0, 15, and 90 min). The other reproductive female (OB4) experienced a 1.6-fold increase in glucocorticoids, likely due to individual differences or past experiences [23,26]. For six of the eight senescent individuals, dermal cortisol concentrations remained below the 2-fold threshold after the stress test. We expected to observe a stress response after the acute stressor in the reproductive life stage, but not in senescent individuals because there would be no adaptive reason for such a response, as their biological systems have begun to shut down as their body degrades [8]. However, two senescent individuals did have a 2-fold increase in response to the stressor (one male, OB2, at 0 min and one female, OB10, at 60 min).

In addition to individual differences within species, there can also be differences between congeneric species. Species’ differences in ecology or physiology can alter how they respond to a stressor [51]. For example, after physiologically stimulating the stress pathway with an injection of ACTH in *Octopus chierchiae*, a female produced dermal cortisol concentration peaks at 15 and 45 min post-injection [28]. A 2-fold increase was also observed in the hummingbird bobtail squid and the stumpy-spined cuttlefish, which are also cephalopods, after an ACTH challenge [28]. In the present study, we used a biological stress test to validate the use of dermal hormone analysis. While both measured a stimulation of the stress axis resulting in elevated glucocorticoids, the stress response to this biological stressor (via creating a stressful biological event) may be dependent on the individual’s experience and sensitivity to handling [23]. Further analysis is needed to examine the differences in initiating stress pathways via a biological stressor versus a physiological stressor.

Age also can influence glucocorticoid production since it can have different roles [52] and functionality during different life stages [53]. Here, we observed varying amounts of dermal glucocorticoid production with the reproductive individuals (~300 ng/mL ethanol) producing significantly lower dermal cortisol concentrations than senescent females (~1000 ng/mL ethanol) and senescent males (~500 ng/mL ethanol). This could be because senescent females were removed from their eggs hours before the experiment to perform the stress test. However, senescent males also had relatively higher levels of dermal cortisol compared to reproductive individuals. This could indicate that they are currently under chronic stress from senescence or have higher levels of cortisol concentrations during this life stage in general. These results may provide evidence that senescence is chronically stressful or that stress physiology may be altered during this life stage.

Additionally, chronic stress has been shown to dampen the response to acute stressors [54]. Perhaps, under higher glucocorticoid concentrations, including significantly higher corticosterone, they reallocate resources such as mobilizing glucose and other nutrients while they cease consuming food, and their bodies degrade. These preliminary results suggest changes in physiology as an octopus becomes senescent. However, more investigation into stress pathways and senescence will be necessary to understand this relationship more fully. While we observed significantly higher reproductive hormones in reproductive than senescent individuals, and vice versa for stress hormones, our total number of individuals is small (*n* = 12), and these results should be seen as instructive and not definitive.

Just as the role of glucocorticoids changes throughout life stages, reproductive hormones are expected to change as well. Cephalopods produce the same reproductive steroid hormones as vertebrates, including estradiol 17-β, progesterone, and testosterone [55]. Here, we determined that in *O. bimaculoides*, dermal estrogen and progesterone concentrations were higher for the reproductive females in comparison to senescent individuals. This was expected since the senescent individuals had already mated, and all females laid eggs. Since all individuals were wild caught, we were unable to determine their exact age or whether the reproductive females had previously mated and were close to being ready to lay eggs. It is known that in *Octopus vulgaris,* estrogen and progesterone levels increase during egg development and preparation of egg laying and then decrease after the reproductive system is primed to lay eggs [16]. In comparison, egg-laying vertebrates produce higher levels of progesterone before laying eggs. In other animals, such as mammals, after the oocyte is ovulated, estradiol begins to decrease while progesterone increases [56].

In *Octopus vulgaris*, reproductive hormones occur at higher concentrations in the sexual organs, suggesting that cephalopods have a reproductive system that, like vertebrates, is under endocrine control [14]. Our study provides some support. In females, dermal testosterone concentrations were significantly higher in reproductive than senescent individuals. Dermal testosterone levels were comparable between reproductive females and males. Yet, dermal testosterone levels in reproductive males only significantly exceeded that of senescent males some 75 min following an acute stressor. A previous study found that the testosterone production of *Octopus maya* males increased as they became mature but decreased as they aged [17]. Similarly, testosterone in *O. maya* females decreases after sexual maturation and slightly increases towards the end of their life [17].

Chronic stressors are known to suppress reproduction, but acute stressors must be particularly severe or repeated to negatively affect reproduction [56]. For example, poorly timed acute stressors could disrupt critical reproductive events, causing a suppression in reproduction [25], and a decrease in estradiol and testosterone concentrations [57]. However, it is unknown if acute stressors have an overall negative effect on reproduction output [25,58]. Our results indicate that acute stressors did not affect dermal reproductive hormones in the short term in all but the two reproductive males that exhibited a 2-fold increase in dermal testosterone at 105 min post-stressor. Their response could be associated with a behavioral response to a threat, such as aggression. Higher levels of testosterone are known to cause aggression in vertebrates [59,60]. Aggression in octopuses has been observed in mating displays [10] and antagonistic signals [61]. Testosterone may play a role as a behavioral mediator of circumstances beyond just modulating sperm production and mating. However, further research is necessary to determine the relationship between testosterone and behavior. In the other individuals, we observed consistent concentrations of the reproductive hormones over time. This consistency demonstrates the ability of the dermal swab method to produce repeatable results within individuals over time.

Finally, when examining the effects of glucocorticoid production on reproductive hormones, we need to be concerned about the possibility of the cross-reactivity of androgens, such as testosterone and cortisol to the EIA antibodies, as observed in previous research [23]. However, we did not observe a direct correlation. For instance, the dermal testosterone concentrations that peaked at 90 min did not correspond to a peak in dermal cortisol concentrations. Dermal cortisol concentrations produced a 2-fold increase at 15 min, demonstrating that these two EIA antibodies did not cross-react with the other steroid. However, biologically, testosterone and cortisol may respond similarly to stress by preparing an animal for aggressive social interactions. For example, high-ranking male baboons (*Papio anubis*) had increased testosterone concentrations along with glucocorticoids [62]. Aggression is known in cephalopods [10,63,64]; however, testosterone has never been measured in relation to this aggression.

## 5. Conclusions

This study exposes changes in dermal glucocorticoid and reproductive hormone concentrations between the reproductive and senescent life stages of *O. bimaculoides*. This is the first time dermal swabs have been used to measure reproductive hormones for any species. We found that reproductive individuals were more likely to have a 2-fold increase in dermal glucocorticoid concentrations; however, most reproductive hormones, apart from testosterone in reproductive males, were not influenced by a biological stressor. This may indicate that reproductive hormones are less susceptible to acute stressors; however, due to the low sample size, further studies are necessary.

This study uses a newer technique to measure hormones by collecting dermal mucus, therefore, we recognize that there are still limitations. The method of swabbing the animal may itself be stressful. Collecting swabs at greater time intervals may help with lowering stress levels compared to collecting swabs at 15 min intervals. However, the stress was minimized by swabbing individuals underwater without moving them to another aquarium. In a previous study, there was found to be no significant difference between swabs taken in tandem underwater or above water [28]. The results in this study expand on that consistency with reproductive hormones lacking the 2-fold change in concentration, which is required to indicate a response from a stressor.

Further, glucocorticoids for cephalopods need to be analyzed using HPLC (high-performance liquid chromatography) to confirm which glucocorticoids are present and measured by our hormone EIA analysis. Our results are based on a small number of individuals, and individual variability is very common when analyzing an animal’s response to stressors [65]. Therefore, we highly recommend further research into cephalopod hormonal changes along with behavioral observations to expand our understanding of cephalopod endocrinology. We also suggest investigations into whether hormone concentrations are constant throughout the entirety of the octopus’s body, to ensure there are no biases for where the mucus is collected.

By studying changes in hormones throughout reproductive and senescent life stages, we can obtain a more complete picture, detailing longitudinal changes of cephalopod reproduction. Long-term studies using swabbing techniques on individuals—from juvenile to senescent—could permit measures of changes in reproductive hormones and glucocorticoids throughout their life. Exploration of diurnal changes of glucocorticoids and sex hormones will also be important, to understand how the time of day for collections may influence hormone measurements. Brief changes in testosterone in reproductive males should also be investigated further to demonstrate whether testosterone modifies individual behavior, such as aggression, along with the relationship between testosterone production and the presence of spermatozoa.

In the wild, senescent individuals do not last long, as they make easy prey. But under human care, they can live to their natural death. As the observer, looking in at the animal in decline may seem a stressful process. Investigating glucocorticoids during the progression of senescence may broaden our understanding and inform us of how to create more favorable welfare conditions under human care.

## Data Availability

These data are available per request made to the corresponding author, S. Chancellor.
